# Preparation and the Biopharmaceutical Evaluation for the Metered Dose Transdermal Spray of Dexketoprofen

**DOI:** 10.1155/2014/697434

**Published:** 2014-02-11

**Authors:** Wangding Lu, Huafei Luo, Zhuangzhi Zhu, Yubo Wu, Jing Luo, Hao Wang

**Affiliations:** National Pharmaceutical Engineering Research Center, China State Institute of Pharmaceutical Industry, Shanghai 201203, China

## Abstract

The objective of the present work was to develop a metered dose transdermal spray (MDTS) formulation for transdermal delivery of dexketoprofen (DE). DE release from a series of formulations was assessed *in vitro*. Various qualitative and quantitative parameters like spray pattern, pump seal efficiency test, average weight per metered dose, and dose uniformity were evaluated. The optimized formulation with good skin permeation and an appropriate drug concentration and permeation enhancer (PE) content was developed incorporating 7% (w/w, %) DE, 7% (v/v, %) isopropyl myristate (IPM), and 93% (v/v, %) ethanol. *In vivo* pharmacokinetic study indicated that the optimized formulation showed a more sustainable plasma-concentration profile compared with the Fenli group. The antiinflammatory effect of DE MDTS was evaluated by experiments involving egg-albumin-induced paw edema in rats and xylene-induced ear swelling in mice. Acetic acid-induced abdominal constriction was used to evaluate the anti-nociceptive actions of DE MDTS. Pharmacodynamic studies indicated that the DE MDTS has good anti-inflammatory and anti-nociceptive activities. Besides, skin irritation studies were performed using rat as an animal model. The results obtained show that the MDTS can be a promising and innovative therapeutic system used in transdermal drug delivery for DE.

## 1. Introduction 

Ketoprofen (KP; (RS)-2-(3-benzoylphenyl)-propionic acid) is nonsteroidal anti-inflammatory drug predominantly used in treatment of rheumatoid arthritis and osteoarthritis. It acts as an anti-inflammatory agent by reversible inhibition of cyclooxygenase 1 and 2 enzymes leading to reduced formation of prostaglandin precursors [1, 2]. Dexketoprofen (DE) is the dextrorotatory enantiomer of ketoprofen. Racemic ketoprofen is used as an anti-inflammatory agent and is one of the most potent *in vitro* inhibitors of prostaglandin synthesis. The effect is due to the (S)-(+)-enantiomer (dexketoprofen), while the (R)-(−)-enantiomer is devoid of such activity. The racemic ketoprofen exhibits little stereoselectivity in its pharmacokinetics [3].

The transdermal drug delivery system (TDDS) offers some advantages compared with its corresponding oral or injectable dosage form applications, such as the provision of steadier drug plasma levels and avoidance of the hepatic first effect [4–6]. Efficacious and safe levels of the drugs through percutaneous absorption are obtained systemically from formulations like transdermal patches, gels, creams, and sprays. Currently, TDDS relies primarily upon occlusive patches, which is now considered to be a mature technology. This system provides controlled release of the drug in patient, enabling a steady blood-level profile, leading to reduced systemic side effects and sometimes improved efficacy over other dosage forms. However, manufacturing of TDDS has historically provided the formulator with some distinct challenges, particularly with the scale-up of multicomponent patches. Additionally, there have also been issues with formulation stability and drug crystallization on longer-term storage. So the negatives of TDDS have been skin irritation, relatively high manufacturing costs, and less-than-ideal cosmetic appearance. Transdermal semisolids such as a gel is an effective alternative to a transdermal patch system. Such a formulation shows a clinically equivalent performance to that of a patch with lesser skin irritation and better compliance [7]. MDTS is a topical aerosol formulated as single phase solution consisting of drug, penetration enhancers, polymers, and solvents. The system developed is a rapid-drying solution containing a volatile component that enables the volume per area of application to be precisely defined. This component also enables the formulation to have uniform distribution on the skin over a defined area after application, without leaving excess vehicle. Hence, this ensures that the dose can be administered in a precise and highly reproducible manner and that aesthetic and transference issues can be avoided. The evaporation of some of the vehicle leads to an increase in concentration of the active drug and hence enhanced partitioning into the stratum corneum [8]. As the MDTS offers advantages of lower skin irritation, greater ease of use, increased dosage flexibility, and a simple manufacturing method, it provides a better alternative to both the patch and gel systems [9, 10].

The objective of this work was to develop a safe MDTS formulation for DE. The *in vitro* drug release was evaluated using hairless mouse skin. The pharmacokinetic and pharmacodynamics characteristics of DE MDTS were evaluated. The developed spray formulations were further evaluated for the performance characteristics like spray pattern, pump seal efficiency test, average weight per metered dose, and content per spray. The skin irritation study was also carried out using rat as an animal model.

## 2. Materials and Methods

### 2.1. Materials

Dexketoprofen ((R, S)-2-(3-benzoylphenyl)propionic acid) with purity of 99.5% was purchased from Huangshi Shixing Pharmaceuticals Co. Ltd. (Huangshi, China). Fenli was purchased from Hubei Anlian Pharmaceutical Co. Ltd. (Wuxue, China). Azone (AZO), isopropyl myristate (IPM), propylene glycol (PG), lauryl lactate (LA), and poly(ethylene glycol) (PEG) 200 were purchased from Merck Chemicals Co. Ltd. (Shanghai, China). Eudragit RL PO was provided by Degussa (Germany). Plasdone S-630 was supplied by International Specialty Products (USA). Kollidone PF 12 and PVP K30 were procured from BASF (Germany). Egg-albumin, xylene, and L-arginine were purchased from Aladdin Industrial Co. (Shanghai, China). Acetic acid was procured from Sino Pharm Chemicals Co. Ltd. (Shanghai, China). All other chemicals and solvents were of analytical reagent grade or chromatography reagent grades.

All the animals used in this study were purchased from the SLAC Laboratory Animal Company Ltd. (Shanghai, China). The animal studies in this study were performed in accordance with the Ethical Guidelines for Investigations in Laboratory Animals and was approved by the National Pharmaceutical Engineering and Research Center.

### 2.2. Solubility Studies

We tested the solubility of DE in different solvent systems (see Table 6). The phosphate saline buffer with various pH levels were prepared according to the Chinese Pharmacopoeia. The solubility of DE was also determined in different penetration enhancers (PE). Excess DE was added into different solvent systems, respectively [11]. The resulting suspensions were shaken at 25 ± 1.0°C for 72 h to get equilibrium. The equilibrated samples were removed from shaker bath and centrifuged for 3 min at 17,800 ×g. The supernatants were taken then filtered (pore size: 0.22 *μ*m) prior to further examination. The sample will be diluted to make sure that the concentration was within the detection range. Saturated concentrations were determined for each solution by HPLC using the method described below.

### 2.3. Formulation Preparation

The MDTS formulations were developed as topical solutions made up of volatile and nonvolatile vehicles containing the drug dissolved in a single phase [12]. The nonvolatile vehicle would be the PE only or the combination of PE and film forming polymer (FFP). The spray system was prepared by incorporating FFP and PE into a solvent system. We used ethanol as the volatile vehicle in this study. The drug application system (Wantong Fixed Quantity Valve System Co. Ltd., Suzhou, China) consisted of a 10 mL container and an actuator with the actuating volume of 100 *μ*L. Formulations were prepared with a series of batches using different PEs or FFPs according to Table 1. The chosen FFPs were based on the following criteria: drying time, cosmetical attractiveness, and outward stickiness.

### 2.4. *In Vitro* Skin Permeation Experiments

We used three animal models for the *in vitro* experiments. They are hairless mice, rat, and porcine. The procedure of the skin was as follows; the dorsal skins of hairless mice or rat were excised after sacrifice by cervical dislocation; porcine skins were obtained from young animals sacrificed at the local slaughter house. Adjacent parts of the same skin were used under different conditions to minimize the skin variability factor. Fresh prepared skins were stored in refrigerator at −20°C without repeatable freeze and thaw cycles. Prior to permeation experiments, skin was thawed and subcutaneous fat, tissue, and capillaries of skin were carefully removed. The skins were washed with normal saline solution and inspected for the integrity by microscope observation. Any skin that had low uniformity was rejected. After cutting into pieces, skin was mounted between the donor and receptor compartment of the Franz diffusion cells with the stratum corneum facing the donor compartment.

The permeation area of Franz diffusion cells was 3.14 cm^2^ and a receiver volume was 7.0 mL. Phosphate buffer saline (PBS) with PH 7.4 was used as the receiver medium. Assembled diffusion cells in triplicate were placed in a transdermal permeation diffusion instrument and maintained isothermally at 32°C. The receptor compartment was stirred with a magnetic stirrer at 220 rpm. The air bubbles that remained in the receptor cell were carefully removed by gentle tilting of the diffusion cells. After the whole system was maintained at 32°C for 2 h, we used micropipette to deliver 100 *μ*L drug liquid precisely and uniformly on the skin. Samples (0.3 mL) were withdrawn at 2, 4, 6, 12, and 24 h for HPLC analysis and were replaced with an equivalent volume. All samples were centrifuged at 17,800 ×g for 3 min and then supernatant was used for analysis.

The cumulative amount *Q* (*μ*g/cm^2^) of DE permeated through skin was calculated by the following equation:

(1) $$\begin{array}{c} Q_{n}=\frac{C_{n}\times V_{0}+\sum_{i=1}^{n-1}\left(C_{i}\times V_{i}\right)}{A}, \end{array}$$

where *A* is the effective area 3.14 cm^2^, *V*_*o*_ is the volume of receptor cell 7.0 mL, *C*_*n*_ is the drug concentration at time point “*n*,” *C*_*i*_ is the DE concentration at time point “*i*,” and *V*_*i*_ is 0.3 mL. The cumulative amount of DE permeated through skin was plotted versus time (h). Each data was expressed as mean ± SD of three determinations.

The steady-state flux value (*J*_ss_) was calculated from the slope of linear portion of cumulative amount permeated-time plots for a zero-order model and expressed as the mass of DE passing across 1 cm^2^ of skin over time.

The enhancement ratio (ER) was determined using the following equation:

(2) $$\begin{array}{c} \text{ER}=\frac{\text{Flux}\;\left(\text{with}\;\text{enhancer}\right)}{\text{Flux}\;\left(\text{without}\;\text{enhancer}\right)\;}. \end{array}$$

### 2.5. Characterization of Developed MDTS Formulations

The qualitative tests performed for the MDTS formulations included the evaluation of spray pattern, effectiveness of pump seal, average weight per metered dose, and content uniformity [13]. The spray pattern was assessed by delivering the spray through the MDTS onto paper. To maintain a constant distance between the point of exit of the spray from the device to the paper, the container was fixed by a fixator for every actuation. The formulation was held at a distance of 5 cm from the paper. The wet part formed was outlined, then the outlined part was clipped from the paper and weighted. Effectiveness of the pump seal was evaluated by pump seal efficiency test. The filled containers under test were placed in the upright position at 30° for 3 days. The containers were weighed before and after the test period. The change in the weight of the container was recorded and the leakage rates were calculated. Average weight per metered dose was measured. The initial weight of the container was recorded; then the container was weighed again after successive deliveries were sprayed from the MDTS. The difference between the initial and final weight of the container divided by the number of delivery sprayed from the containers was used to determine the average weight per metered dose. The DE content per spray was determined by actuating designed sprays in a beaker containing methanol. Then the drug content was analyzed by HPLC.

The drug administration area of each pump was calculated by the following equation:

(3) $$\begin{array}{c} A_{x}=\frac{W_{o}\times A_{o}}{W_{x}}, \end{array}$$

where *W*_*o*_ and *A*_*o*_ are the known weight and area, respectively, of the paper we clipped from the paper, *W*_*x*_ is the weight of the paper after certain MDTS actuation, and *A*_*x*_ is the area of certain pump. Taking paper with area of 10 cm × 10 cm and weighted 0.8166 g as a sample, *W*_*o*_ is 0.8166 g and *A*_*o*_ is 100 cm^2^.

The pump seal efficiency was calculated by the following equation:

(4) $$\begin{array}{c} \text{Leakage}\;\text{rate}=\frac{\left(W_{\text{before}\;\text{test}}-W_{\text{after}\;\text{test}}\right)}{W_{\text{before}\;\text{test}}}; \end{array}$$

*W*_before  test_ and W_after  test_ were the weight of the container before and after the test period, respectively.

Average weight per metered dose was measured by the following equation:

(5) $$\begin{array}{c} W_{n-m}=\frac{\left(W_{n}-W_{m}\right)}{\left(m-n\right)}\times 100\%, \end{array}$$

where *W*_*n*_ and *W*_*m*_ were the weight of the “*n*” and “*m*” actuation times, respectively. *W*_*n*−*m*_ was the average weight per metered dose during the “*n*” to “*m*” actuation times.

### 2.6. Pharmacokinetic Study

Healthy female Sprague-Dawley rats weighing 240 ± 20 g were used in this study. The animals were housed four per cage in laminar flow that was maintained at 22 ± 2°C and 50–60% relative humidity. The animals were kept in these facilities for at least 1 week prior to the experiment and were fasted for at least 24 h before commencing the experiment. Before administration, the abdominal hair was shaved using an electric clipper carefully and allowed to heal for 24 h. The animals were divided into three groups randomly with four animals in each group [14]. The first group was applied with DE MDTS. A dose of 20 *μ*L solution containing 1.4 mg DE was delivered to the fixed area (2 cm × 2 cm) on the shaved skin of rats by a micropipette. The second group was treated with Fenli; it was an oral tablet product of DE sold in Chinese market. The drug was dissolved in ethanol, and 3 mL drug solution containing 1.4 mg DE was delivered by intragastric injection. The third group received 0.3 mL DE solution containing 1.4 mg DE via the tail vein. The injection solution was prepared as follows: L-arginine was dissolved by water for injection and then DE was added. After decolorizing with 0.1% active carbon and filtrating by 0.22 *μ*m membrane, this solution was sterilized at 115°C for 30 min in a sealed ampoule. Blood samples were collected into heparinized tubes at the scheduled sampling time via retroorbital plexus using a sterilized glass capillary tube. After centrifugation for 3 min at 17,800 ×g, the separated serum of 100 *μ*L was transferred into another neat tube and frozen at −20°C until the determination of DE concentration by UPLC-MS/MS analysis.

The pharmacokinetic parameters such as peak plasma concentration during the dosing period (*C*_max⁡_) and time of peak plasma concentration (*T*_max⁡_), the area under the profile (AUC_0→*t*_), the half-life of elimination from plasma (*t*_1/2_), and the mean residence time (MRT) were calculated by noncompartment analysis following transdermal application using DAS 2.0 software.

Absolute bioavailability *F*(%) was calculated from the following equation:

(6) $$\begin{array}{c} F\left(\%\right)=\frac{\text{AU}{\text{C}}_{\text{optimal}\;\text{formulation}}}{\text{AU}{\text{C}}_{i\cdot v}}\times 100\%. \end{array}$$

Relative bioavailability *F*(%) was calculated from the following equation:

(7) $$\begin{array}{c} F\left(\%\right)=\frac{{\text{AUC}}_{\text{optimal}\;\text{formulation}}}{{\text{AUC}}_{\text{oral}}}\times 100\%. \end{array}$$

In this study, the dosage we give to each rat was 1.4 mg, AUC_*i*·*v*_, AUC_oral_, and AUC_optimal  formulation_ were the AUC_0→*t*_ for intranvenous, oral, and transdermal routs, respectively.

### 2.7. Egg-Albumin Induced Paw Edema in Rats

Healthy female Sprague-Dawley rats weighing 200 ± 20 g were divided into three groups (*n* = 6) [15]. Before treatment, the circumference of ankle joint of the right hind paw was measured as the zero time circumference. 2 h after intragastric injection of Fenli (7.0 mg/kg based on DE) and transdermal administration of DE MDTS (see Table 8) (7.0 mg/kg based on DE), peripheral inflammation was induced by intraplantar injection of 10% egg-albumin solution (0.1 mL) into the middle of the plantar surface of the right hind paw. The remaining group without drug treatment was used as the control group. Then the circumference of the ankle joint of the right hind paw was measured at 30 min, 1 h, 2 h, 3 h, and 6 h after injection of the 10% egg-albumin solution.

For the study of egg-albumin induced paw edema in rats, the swelling degree was calculated from the following equation:

(8) $$\begin{array}{c} \text{Swelling}\;\text{degree}=C_{2}-C_{1}; \end{array}$$

*C*_1_ is circumference before administration and *C*_2_ is circumference after administration.

### 2.8. Xylene-Induced Ear Swelling in Mice

The mice weighing 20 ± 2 g were placed into three random groups (*n* = 9), and each animal received 50 *μ*L xylene on the anterior and posterior surfaces of the right ear lobe 1 h after intragastric injection of Fenli (7.0 mg/kg based on DE) and transdermal administration of DE MTDS (7.0 mg/kg based on DE); the left ear was considered as a control. The remaining group without drug treatment was used as the control group. Two hours later, the animals were sacrificed by cervical dislocation and both ears were sampled. Circular sections were taken, using a cork borer with a diameter of 8 mm, and weighed immediately. The degree of ear swelling was calculated based on the weight of the left ear without application of xylene [16].

For the study of egg-albumin induced paw edema in rats, the swelling degree was calculated from the following equation:

(9) Swelling  degree  (SD)  =weigh  of  right  ear−weigh  of  left  ear,Inhibition  rate=(SD1−SD2)SD1×100%,

with SD_1_, SD_2_ of the control group and SD2, SD of the test group.

### 2.9. Acetic Acid-Induced Abdominal Constriction in Mice

Mice weighing 20 ± 2 g were placed into three groups (*n* = 9) and given intraperitoneal injections of 0.25 mL/10 g body weight of 1.5% acetic acid solution in saline 1 h after intragastric injection of Fenli (7.0 mg/kg based on DE) and transdermal administration of DE MDTS (7.0 mg/kg based on DE). The remaining group without drug treatment was used as the control group. Writhing was characterized by a wave of contraction of the abdominal musculature followed by the extension of the hind limbs. The frequency of writhing observed was recorded 20 min after the injection of acetic acid [17].

For the study of acetic acid-induced abdominal constriction in mice, the pain-inhibition rate was calculated from the following equation:

(10) $$\begin{array}{c} \text{Pain-inhibition}\;\text{rate}=\frac{\left(W_{c}-W_{t}\right)}{W_{c}}\times 100\%; \end{array}$$

*W*_*c*_ is writhing count of the control group; *W*_*t*_ is writhing count of the test group.

### 2.10. Skin Irritation Study

Draize patch test was carried out using rat as the animal model. Healthy female Sprague-Dawley rats weighing 220 ± 20 g were used in this study. The abdominal hair was shaved using an electric clipper carefully and allowed to heal for 24 h. The animals were divided into two groups randomly with six animals in each group. The first group was treated with the optimized formulation spraying on the patch of preshaved skin and occluded with adhesive tapes. The second group was only occluded with adhesive tapes without drug treatment. Then the resulting reactions such as erythema and edema were scored after 24 h [18].

### 2.11. *In Vitro* HPLC Analysis of DE

The samples of DE *in vitro* experiments were analyzed using an HPLC system consisting of a system controller (SCL-10 ATVP; Shimadzu, Japan), a binary pump (LC-10 ATVP, Shimadzu), a UV-VIS detector (SPD-10 AVP, Shimadzu), a column oven, and an autoinjector (SIL-10A, Shimadzu). The separation method was under the following conditions: *C*_18_ reversed phase analytical column (4.6 × 150 mm^2^, 5 *μ*m, Shim-pack VP-ODS). The mobile phase was 60 : 40 (v/v) methanol-ammonium acetate buffer (0.05 M, pH 4.0), column temperature of 40°C, UV detective wavelength of 257 nm, flow rate of 1.0 mL/min, and injection volume of 10 *μ*L. The data were acquired and analyzed by Shimadzu Class-VP chromatography software. There was no interference from skin and a well-separated peak was detected at the retention time of 9.1 ± 0.1 min with the sensitivity of 0.02 *μ*g/mL. The peak area correlated linearly with DE concentration in the range from 1 to 500 *μ*g/mL.

### 2.12. *In Vivo* UPLC-MS/MS Analysis of DE

The analyte was recovered from plasma samples by liquid-liquid extraction (LLE) after thawed thoroughly at room temperature [19]. A 100 *μ*L aliquot of plasma, 10 *μ*L ibuprofen (1 *μ*g/mL) as internal standard (IS), and 10 *μ*L 0.1 HCl (1 mol/L) were pipetted into 1.5 mL centrifuge tubes. Samples were extracted using 1 mL ethyl acetate and the tubes were vortexed for 2 min prior to centrifugation at 17,800 ×g for 3 min. Then 800 *μ*L supernatant from each centrifuge tube was pipetted into sample insert and evaporated to dryness completely at 40°C with a vacuum centrifugal concentrator (miVac DUO, Genevac). Samples were then reconstituted with 200 *μ*L 50 : 50 (v/v) methanol-water, the sample vials were vortexed for a further 1 min and centrifuged at 17,800 ×g for 3 min, and then the supernatants were used for analysis. Analysis of DE and plasma was performed with UPLC-MS/MS system equipped with a system controller (SCL-10 ATVP; Shimadzu), a binary pump (LC-10 ATVP; Shimadzu), a UV-VIS detector (SPD-10 AVP, Shimadzu), a column oven, and an auto injector (SIL-10A; Shimadzu) with an electrospray ionization (ESI) interface. The UPLC separation method was under the following conditions: *C*_18_ reversed phase analytical column (Shim-pack XR-ODS) (2.0 I.D. × 75 mm^2^, 1.6 *μ*m), mobile phase of methanol and 10 mM ammonium acetate buffer, column temperature of 40°C, detective wavelength of 257 nm, flow rate of 0.3 mL/min, and injection volume of 5 *μ*L (see Table 5). A gradient elution was carried out using a mobile phase consisted of a mixture of A (10 mM ammonium acetate buffer) and B (methanol) at a flow rate of 0.3 mL/min according to the following multistep gradients shown in Tables 2 and 3.

The MS/MS conditions were set as follows: the ionization method was ESI, which was operated in positive single ion monitoring (SIM+) mode. Nitrogen was used as the nebulizer and desolvation gas with the flow rate of 3 and 15 L/min, respectively. The capillary temperature and voltage were set at 400°C and 3.0 kV. Desolvation temperature was set at 400°C. Quantification was performed using multiple reaction monitoring mode with transition of m/z 205.10 → 161.00 for DE and m/z 253.10 → 109.10 for IS. The data were acquired and analyzed by Shimadzu Labsolutions software. The retention times were 2.3 ± 0.1 and 2.8 ± 0.1 min for DE and IS, respectively.

The analytical column and mobile phase used for the assay provided a clear separation between DE and internal standard. There was no interference from any endogenous material. The validation of analytical method for DE showed that the method was precise and accurate with a linear range of 0.05–80 *μ*g/mL. The mean recovery of DE from plasma in the quality control samples (0.1, 10, and 64 *μ*g/mL) was 80.26 ± 3.67%, 72.13 ± 4.21%, and 62.34 ± 2.54%, respectively. The intraday and interday assay coefficients of variation were 2.21% and 2.98%, demonstrating good reproducibility.

### 2.13. Statistical Analysis

Data were presented as mean value ± standard deviation (SD). Statistical data were analyzed by Student's *t-*test or one-way analysis of variance using SPSS version 16.0. The level of significance was set at *P* < 0.05.

## 3. Results and Discussion

Pharmacokinetic differences between the enantiomers could be caused by chiral inversion. Ketoprofen underwent unidirectional chiral inversion from the R- to the S-enantiomer. The extent of inversion varied considerably between species. The extent of inversion was not affected by the dose rate [20, 21]. Administration of racemic ketoprofen instead of a pure enantiomer had an influence on the enantiomer concentration ratio in plasma [22, 23], while inversion was usually unidirectional from R (+) to S (+) KTP except in CD-1 mice where a substantial bidirectional inversion was noted [24].

As results shown in Table 4, the solubility of the screened receptor medium was PBS (pH 7.4) > 40% PEG > PBS (pH 7.0) > PBS (pH 6.5) > 30% PEG > 20% PEG. To ensure stable collection conditions, PBS with pH 7.4 was used as receptor median to remain a “sink condition.” Solubility of DE in different PEs might be a critical factor for the PE screening. The solubility of DE in the chosen PE was PG > IPM> LA> AZO. Based on the hypothesis that the PE would act as a “vehicle” for the drug, the more the drug is solubilized in the vehicle, the higher transdermal flux will be reached [25–27].

The film formed by the formulation incorporating FFP was transparent and cohesive. The volatile solvent ethanol in the formulation evaporated quickly leaving behind a thin film that adhered to the skin. By varying the ratio of the FFP, based on the visualization of the film formed, we chose 5% as the content of FFP. All formulations no matter including only PE or containing both FFP and PE were with appearances of clarity.

For the* in vitro* skin permeation experiments, the effects of FFP, PE, and DE concentration and the screened PE content on skin permeation were investigated to optimize the DE MTDS formulation.

**Figure 1:**
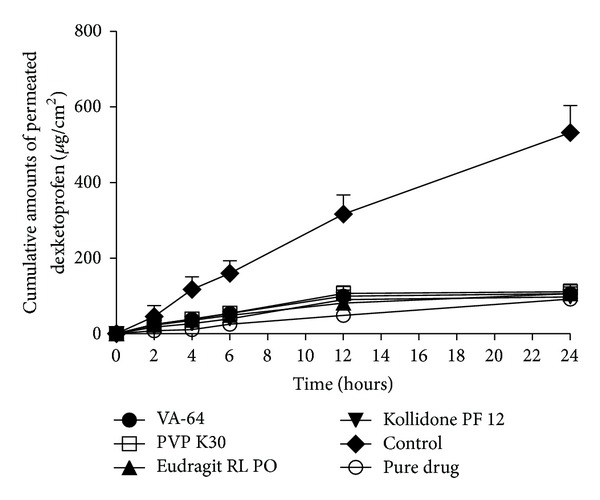
Percutaneous permeation profiles of DE MTDS containing different film forming polymers (mean ± SD; *n* = 3).

The results of F1 to 4 were shown in Figure 1, and the control group was F9 described in Table 1. In order to confirm the permeation enhancement of the ethanol evaporation, we added the pure drug group, which meant that the equal amount of drug to other groups was uniformly put on the skin. The transdermal permeation profiles of formulations containing different FFPs did not show significant difference. The formulation including FFP reduced the permeation of DE significantly compared with the control group, indicating that the FFP would inhibit the transdermal delivery of DE. The significant difference between the control and pure drug group, indicating the evaporation of ethanol, would enhance the permeation effect.

**Figure 2:**
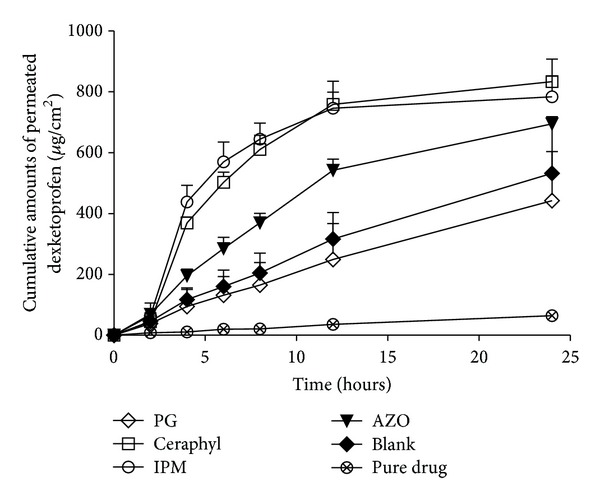
Percutaneous permeation profiles of dexketoprofen MTDS containing different penetration enhancers (mean ± SD; *n* = 3).

The results of F5 to 8 were shown in Figure 2. The control and pure drug group was the same as the one in Figure 1. As seen from the figure, the transdermal flux of them was LA > IPM > AZO > PG. LA and IPM showed comparable transdermal flux without significant difference at this concentration level. Though PG had the greatest solubility for DE, its transdermal flux was the lowest. This might attribute to the fact proven by Trottet that PG would permeate through the skin and might carry the drug with it, as shown by correlations *in vitro* between the permeation of both PG and the drug [28]. As the PG permeated through the skin, the “drug reservoir” in the skin would not be formed. Besides, the investigation of influence of penetration enhancer on drug permeation from volatile formulations by Hadgraft reconfirmed the conclusion stated by Trottet. In addition, Hadgraft presented that, after administration, IPM remained in the skin to form a “patchless drug reservoir” instead of permeating through the skin like PG did [29]. The AZO group showed a relatively low tansdermal flux compared with the IPM and LA group; to some extent, it indicated that the solubility of DE in PE was a critical fact determining the transdermal flux [30, 31]. The transdermal flux of the control group is much higher compared with the group containing pure drug. This might attribute to the fact that the evaporation of ethanol could increase the thermodynamic energy of drug. Besides, ethanol also can be used as permeation enhancer in some cases [32].

**Figure 3:**
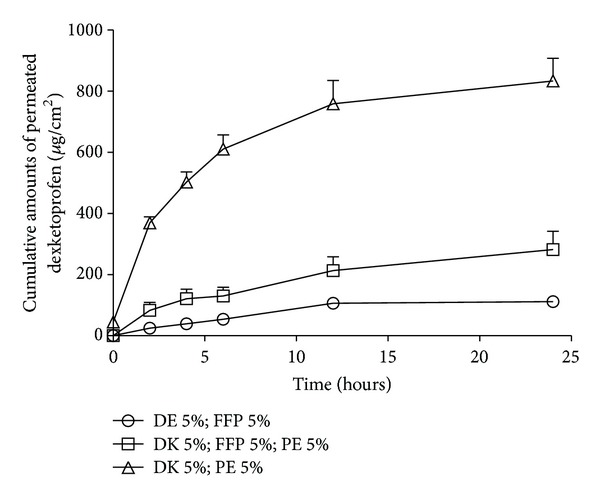
Percutaneous permeation profiles of dexketoprofen MTDS (F3, F6, and F10, resp.) (mean ± SD; *n* = 3).

As the results shown in Figure 1, we can see that the transdermal flux of the formulations incorporating different FFPs did not show significant difference. We choose PVP K30 as the FFP to investigate the formulation only containing PE or FFP and formulation containing both PE and FFP. Results shown in Figure 3 reconfirmed the fact that FFP would inhibit the transdermal permeation of DE. Based on these results, we decided that the compositions of the formulation were DE, PE, and FFP.

**Figure 4:**
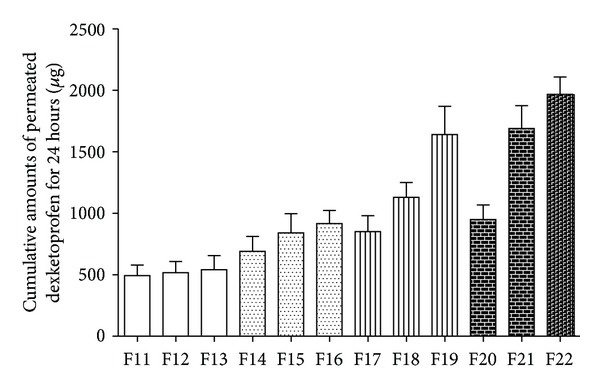
Cumulative amounts of permeated DE for 24 hour, *Q*_24 h_ (*μ*g/cm^2^) of F11 to F22 (mean ± SD; *n* = 3).

By differing the DE and LA concentration, the percutaneous permeation profiles of each formulation were shown. As the results shown in Figure 4, the transdermal flux of DE did not show significant difference with the increasing content of LA when DE was at a relatively low concentration 3%, while the transdermal flux responded positively with the increasing ratio of LA when the concentration of DE was 5%, 7%, and 10, respectively. Based on the hypothesis that the PE would act as “vehicle” for the drug, when at a relatively low drug concentration 3%, there were enough vehicles prepared for the drug to cross the skin even if the LA was only 5%. So increasing the LA concentration would not affect the percutaneous permeation behavior. For the formulations including 5% or 7% DE, the transdermal flux responded positively with the ratio of LA. This might attribute to the reason that with more LA, more drug would be solubilized; as a result the transdermal flux increased. Though the transdermal flux was indeed responding positively with the increasing level of LA when DE was 10%, the increased percutaneous drug amount caused by F22 compared with F21 was lower than that caused by F21 compared with F20. This could be explained that, after the volatile solvent evaporated, the LA was not fast enough to carry the drug into the skin; then the drug crystallized outside the skin. Further investigations were needed to illustrate it.

**Figure 5:**
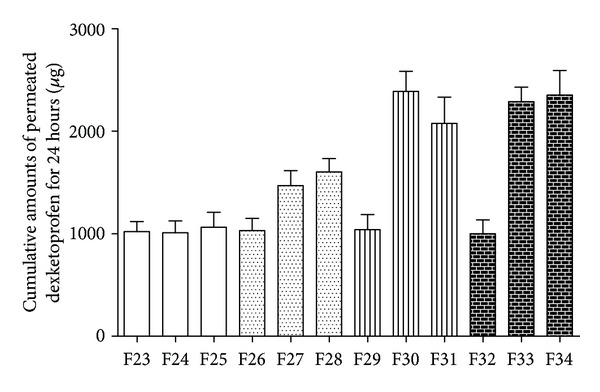
Cumulative amounts of permeated DE for 24 hour, *Q*_24 h_ (*μ*g) of F23 to F34 (mean ± SD; *n* = 3).

Since the enhancement ratio (ER) of IPM and LA did not show significant difference, we also investigated the formulations with various drug and IPM concentrations. As seen in Figure 5, the transdermal flux of DE did not show significant difference with the increasing content of IPM when DE was at a relatively low concentration 3%, while the percutaneous drug amount was higher than the formulation containing LA with 3% DE. To some extent, it revealed that the loading capability of IPM was stronger than LA, which needed further investigation. With a relatively higher IPM level at 10%, the transdermal flux did not improve much compared with the formulations containing 7% IPM when the DE was 5%, 7% and 10%, respectively. This might attribute to the fact that 7% IPM would provide sufficient vehicle for the drug when DE is at the concentrations of 5%, 7%, and 10%. When DE is at a relatively higher level 10%, the transdermal flux did not show significant difference compared with 7%; this might attribute to the same reason demonstrated above. Based on the DE concentration, amount of PE, and skin permeation behavior, we chose formulation containing 7% (w/w, %) DE, 7% (v/v, %) isopropyl myristate (IPM), and 93% (v/v, %) ethanol as the optimized formulation.

**Figure 6:**
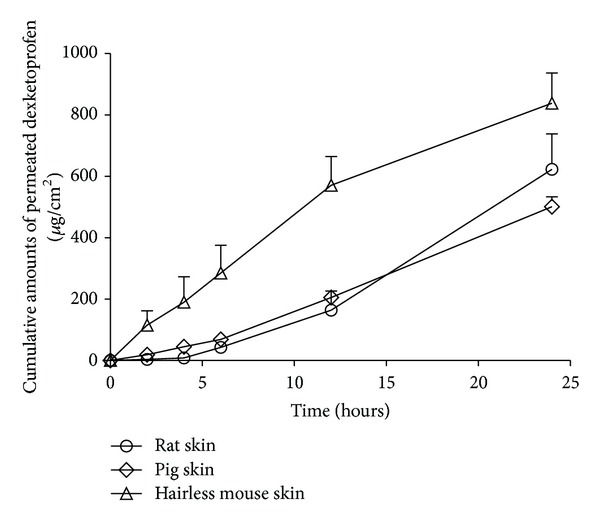
Percutaneous permeation profiles of F30 in different *in vitro* models (mean ± SD; *n* = 3).

A key goal in the design and optimization of dermal or transdermal dosage forms lied in understanding the factors that determine a good* in vivo* performance. Variations in methodology used with a specific skin model, such as type of diffusion cells, skin temperature, receiver media, application dose, and diffusion area, would all significantly affect data. Since the human skin availability was limited, a wide range of animal models had been suggested as a suitable replacement for human skin and had been used to evaluate percutaneous permeation of molecules. The histological and biochemical properties of porcine skin had been repeatedly shown to be similar to human skin [33–36]. Skin of rodents (mice, rats, and guinea pigs) was the most commonly used in *in vitro* and *in vivo* percutaneous permeation studies due to their small size, uncomplicated handling, and relatively low cost. There are a number of hairless species (nude mice and hairless rats) in which the absence of hair coat mimics the human skin better than hairy skin [37]. In these animals there is no need for hair removal (clipping or shaving) prior to the experiment, thus avoiding the risk of injury to cutaneous tissue. Other models have a disadvantage of an extremely high density of hair follicles and require hair removal. Since both issues may affect percutaneous absorption of molecules, hairy rodent skin is usually not used in *in vitro* permeation studies, although *in vivo* studies are still performed on these species. In this study we used the hairless mouse as the *in vitro* animal model. We also investigated the percutaneous behavior of the optimized formulation in other animal models to study the corelation among these three models (see Table 7). As results shown in Figure 6, the transdermal DE amount of the hairless mouse group was about twofold of the porcine skin group. The *J*_ss_ (*μ*g/(cm^2^·h)) between hairless mouse group and porcine group did not show significant difference.

For the characterization of the developed MDTS formulation, we evaluated the drug administration of each pump. The results indicated that this MDTS formulation showed uniform spray pattern. No leakage was observed from the MDTS containers when placed in the upright position at 30° for 3 d. Content uniformity was assessed for 1th, 5th, 10th, 20th, and 40th doses and the results indicated that the MDTS can perform uniform content per actuation (see Table 9). Average weight per metered dose is an important quantitative parameter to be evaluated. And the DE content per spray was also determined. The results indicated that the DE MDTS showed reproducible amounts of the formulation per actuation.

**Figure 7:**
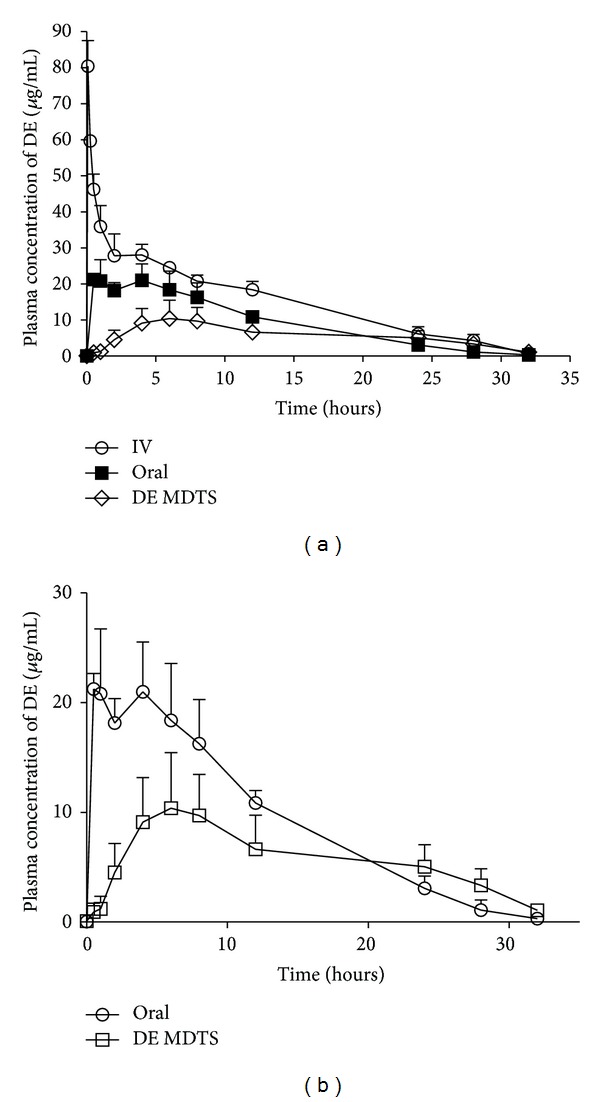
*In vivo* absorption profiles of DE after IV, oral, and transdermal administration in rats (mean ± SD; *n* = 4).

Rodents have a thinner stratum corneum and higher hair follicles density than human skin, so it may overestimate the permeability of drugs in human when using rodent's skin as model. However, the recent research indicated that Sprague-Dawley rat was a useful model for predicting human skin permeability with low interindividual variations and similar permeating rate (with twofold difference) [38]. In this experiment, the pharmacokinetic studies were conducted in rats for intravenous, transdermal, and oral routs. The mean plasma concentration-time of DE after IV, transdermal, and oral administration was presented in Figure 7. A summary of the pharmacokinetic parameters was shown in Table 10. As seen in Figure 7, the plasma concentration of IV group decreased promptly after drug administration. For the oral and transdermal administration group, the plasma DE concentrations increased to the peak level after administration; thereafter, the plasma concentrations gradually declined. The peak plasma concentration of DE MDTS group was 11.23 *μ*g/mL at 6.5 h, which decreased gradually to 5.05 *μ*g/mL at 24 h. For the oral administration group, the peak plasma concentration was 23.88 *μ*g/mL at 1.5 h, while it deceased to 3.07 *μ*g/mL at 24 h. The result indicated that DE MDTS showed a more sustainable plasma concentration-time profile compared with oral administration group. The absolute bioavailability of DE MDTS was 37.45%. And the relative bioavailability was 62.19%.

**Figure 8:**
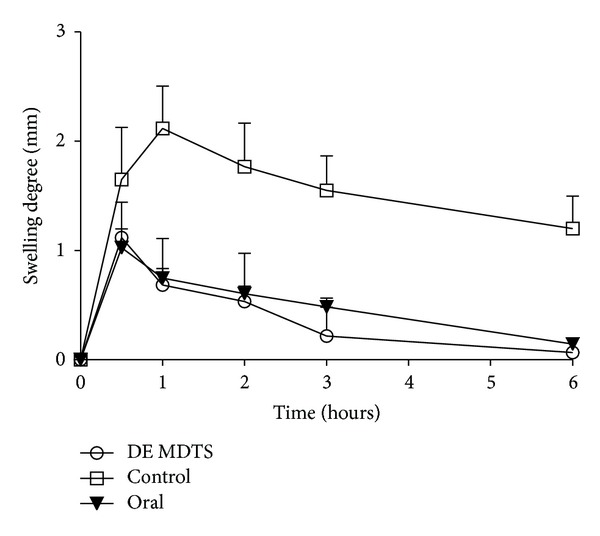
Anti-inflammatory effects of DE MDTS and Fenli on egg-albumin induced rat hind paw edema (mean ± SD; *n* = 6).

The experiment involving egg-albumin induced paw edema in rats was used to compare the anti-inflammatory performances of DE MDTS and Fenli. The hind paw edema-time curve was shown in Figure 8. After stimulation by the short-acting inflammatory agent, egg-albumin, the hind paw exhibited marked swelling at 0.5 h, which then decreased gradually to recovery over the next few hours for the DE MDTS and Fenli group. For the control group, the swelling degree reached its peak level at 1 h then decreased gradually over the next few hours. At the end-point 6 h of observing, the swelling degree of the Fenli, DE MDTS, and control group was 0.00 ± 0.02, 0.10 ± 0.11, and 0.87 ± 0.21, respectively. As far as comparison of the Fenli with the DE MDTS group was concerned, the former exhibited less edema from 1 to 3 h (*P* < 0.05), while both groups showed a comparable anti-inflammatory effect at 6 h.

To evaluate the anti-inflammatory effect of DE MDTS in other experiment animals, mice were selected and the xylene-induced ear swelling test was carried out. Vascular reactions always occur in the early stage of the inflammation process. In this stage, the inflamed tissue produces many kinds of inflammatory mediators, such as prostaglandins (PGs), bradykinin, and histamine [28]. These substances act on the endothelial cells of the blood vessels, resulting in the shrinkage of the endothelial cells and the formation of endothelial cell gaps. In addition, other mechanisms, such as leukocyte-mediated endothelial cell injury, also lead to enhanced local vasopermeability. One hour after smearing with xylene, the degree of swelling in the Fenli group, DE MDTS group and control group was 5.13 ± 0.68, 5.86 ± 1.76, and 16.63 ± 1.57, respectively. As can be seen from Table 11, the inhibition rates for the Fenli group and DE MS group were 69.15% and 64.76%, respectively. The difference of anti-inflammatory effect between these two groups might be the result of different pharmacokinetic characteristics.

The acetic acid-induced abdominal constriction experiment was used to evaluate the antinociceptive effect of DE MDTS, in comparison with Fenli. As shown in Table 12, the writhing count of Fenli group, DE MDTS group, and control group was 5.83 ± 1.32, 8.13 ± 1.78, and 24.33 ± 3.08, respectively. The pain-inhibition rate of the Fenli and DE MDTS group was 76.04% and 70.69%, respectively. Both groups had significantly restrained the writhing responses of the mice.

No obvious redness and swelling were found on skin in the primary skin irritation studies with the optimized formulations on the rat skin hence thought to be a skin nonirritant application based on present study in this animal model.

## 4. Conclusions 

A novel transdermal drug delivery system was designed and evaluated in *in vitro* and *in vivo* studies. The effects of FFP, PE, and DE concentration and the content of the screened enhancer on skin permeation behavior were investigated to find out the optimized formulation. The final formulation provided satisfactory skin permeation with an appropriate combination of DE and IPM content. The pharmacokinetic parameters of the optimal formulation indicated that the optimized formulation showed a more sustainable plasma-concentration profile compared with the commercial product, Fenli. The pharmacodynamic studies indicated that DE MDTS had a significant anti-inflammatory and antinociceptive effects. Besides, characterization of DE MDTS indicated that it could deliver reproducible amounts of the formulation per actuation. No obvious erythema or edema were found to occur in the primary skin irritation studies of the optimized formulations on the rat. From the results obtained in the present work, it can be concluded that the MDTS can be a promising and innovative therapeutic system for the transdermal drug delivery of DE.

## Figures and Tables

**Table 1:** Composition of investigated formulation for excipients screening.

Ingredients	F1	F2	F3	F4	F5	F6	F7	F8	F9	F10
DE (%w/w)	5	5	5	5	5	5	5	5	5	5
PE (%v/v)	—	—	—	—	5	5	5	5	0	5
FFP (%w/w)	5	5	5	5	0	0	0	0	0	5
Absolute alcohol	Add to 10 mL									

*The FFPs used in F1 to F4 were Eudragit RL PO, Plasdone S-630, PVP K30, and Kollidone PF 12, respectively; the PEs used in F5 to F8 were AZO, IPM, LA and PG, respectively; F9 was used as the control group; F10 contained both PE and FFP.

**Table 2:** Composition of the formulation for optimization (a).

Ingredients	F11	F12	F13	F14	F15	F16	F17	F18	F19	F20	F21	F22
DE (%w/w)	3	3	3	5	5	5	7	7	7	10	10	10
LA (%v/v)	5	7	10	5	7	10	5	7	10	5	7	10
Absolute alcohol	Add to 10 mL											

**Table 3:** Composition of the formulation for optimization (b).

Ingredients	F23	F24	F25	F26	F27	F28	F29	F30	F31	F32	F33	F34
DE (%w/w)	3	3	3	5	5	5	7	7	7	10	10	10
IPM (%v/v)	5	7	10	5	7	10	5	7	10	5	7	10
Absolute alcohol	Add to 10 mL											

**Table 4:** Skin irritation score scale.

Grading	Description of irritant response
0	No reaction
+	Weakly positive reaction (usually characterized by mild erythema across most of the treatment site)
+ +	Moderate positive reaction (usually distinct erythema possibly spreading beyond the treatment site)
+ + +	Strongly positive reaction (strong, often spreading erythema with edema)

**Table 5:** Gradient conditions for UPLC.

Inlet	Time (min)	Flow rate (mL/min)	A (%)^a^	B (%)^b^
1	Initial	0.3	60	40
2	0.5	0.3	40	60
3	2	0.3	5	95
4	3	0.3	5	95
5	3.2	0.3	60	40
6	4	0.3	60	40

^a^10 mM ammonium acetate buffer; ^b^methanol.

**Table 6:** Solubility of DE in different solvents (*n* = 6).

Solvents	Solubility (mg/mL)
PBS (pH 6.5)	33.73 ± 0.20
PBS (pH 7.0)	63.26 ± 0.99
PBS (pH 7.4)	78.89 ± 0.06
20% PEG	12.53 ± 3.33
30% PEG	28.76 ± 0.03
40% PEG	76.48 ± 0.13
IPM	142.92 ± 5.51
PG	252.61 ± 5.34
AZO	22.37 ± 3.32
LA	47.79 ± 5.61

**Table 7:** Percutaneous behavior results of three different* in vitro* animal models (mean ± SD; *n* = 3).

Groups	*Q*_24_ (µg/cm^2^)	*J*_ss_ (µg/(cm^2^·h))
Hairless mouse skin	838.17 ± 98.48	33.59 ± 6.16
Rat skin	622.59 ± 115.85	36.34 ± 7.24
Porcine skin	484.37 ± 30.73	25.33 ± 1.92

**Table 8:** Evaluation of DE MTDS administration area (mean ± SD; *n* = 6).

No.	Weight (g)	Area (cm^2^)	Mean area (cm^2^)
1	0.1549	17.30	
2	0.1536	17.16	
3	0.1527	17.06	17.20 ± 0.18
4	0.1516	16.93	
5	0.1558	17.40	
6	0.1552	17.34	

Student's* t-*test and one-way analysis of variance (ANOVA) to determine the level of significance, *P* > 0.05.

**Table 9:** Evaluation of per actuation content for DE MDTS (mean ± SD; *n* = 6).

Actuation times	Weight per pump (mg)	DE for per pump (mg)
0-1th	91.13 ± 0.09	6.29 ± 0.09
1–5th	91.75 ± 0.11	6.31 ± 0.13
5–10th	90.06 ± 0.13	6.30 ± 0.12
10–20th	91.35 ± 0.14	6.27 ± 0.14
20–40th	91.20 ± 0.10	6.32 ± 0.12

Student's *t*-test and one-way analysis of variance (ANOVA) to determine the level of significance, *P* > 0.05.

**Table 10:** Pharmacokinetic parameters of dexketoprofen after IV, oral, and transdermal administration in rats (mean ± SD; *n* = 4).

Parameters	IV	Oral	DE MDTS
AUC_0→*t*_ (ng·mL^−1^·h)	494.442 ± 20.788	296.662 ± 44.321	185.137 ± 67.792
MRT_0→*t*_ (h)	9.311 ± 1.113	8.758 ± 0.682	13.459 ± 0.684
*t*_1/2_ (h)	5.555 ± 2.018	3.778 ± 1.67	7.688 ± 2.546
*t*_max⁡_ (h)	0.083 ± 0	1.5 ± 1.683	6.5 ± 1
*c*_max⁡_ (ng/mL)	80.43 ± 8.67	23.875 ± 4.632	11.231 ± 4.676

**Table 11:** Anti-inflammatory effects of DE on xylene-induced ear swelling mice (mean ± SD; *n* = 9).

Group	Swell degree (mg)	Inhibition rate (%)
Fenli	5.13 ± 0.68	69.15
DE MDTS	5.86 ± 1.76	64.76
Control	16.63 ± 1.57	

**Table 12:** Antinociceptive effects of DE on acid-induced abdominal constriction in mice (mean ± SD; *n* = 9).

Group	Writhing count	Pain-inhibition rate (%)
Fenli	5.83 ± 1.32	76.04
DE MTDS	7.13 ± 1.78	70.69
Control	24.33 ± 3.08	
